# Decompressive craniectomy following subarachnoid hemorrhage: A prospective Swedish multicenter study

**DOI:** 10.1016/j.bas.2025.104218

**Published:** 2025-02-20

**Authors:** Bryndís Baldvinsdóttir, Erik Kronvall, Elisabeth Ronne-Engström, Per Enblad, Paula Klurfan, Johanna Eneling, Peter Lindvall, Helena Aineskog, Steen Friðriksson, Mikael Svensson, Peter Alpkvist, Jan Hillman, Ola G. Nilsson

**Affiliations:** a Department of Clinical Sciences, Neurosurgery, Lund University, Lund, Sweden; b Department of Neuroscience, Section of Neurosurgery, Uppsala University, Uppsala, Sweden; c Department of Clinical Neuroscience, University of Gothenburg, Gothenburg, Sweden; d Department of Clinical Sciences, Linköping University, Linköping, Sweden; e Department of Clinical Sciences, Umeå University, Umeå, Sweden; f Department of Clinical Neuroscience, Karolinska Institutet, Stockholm, Sweden

**Keywords:** Nationwide, Aneurysmal subarachnoid hemorrhage, Decompressive craniectomy, Adverse events, Glasgow outcome scale extended

## Abstract

**Introduction:**

Decompressive craniectomy (DC) in patients with severe aneurysmal subarachnoid hemorrhage (aSAH) can be a life-saving procedure. The aim of this nationwide prospective study was to investigate the use of DC in aSAH patients in Sweden.

**Research question:**

To explore the risk factors and functional outcome associated with DC in patients with aSAH.

**Material and methods:**

Patients treated for aSAH at all neurosurgical centers in Sweden during a 3.5-year period (2014–2018) were prospectively registered. Clinical, radiological and treatment-related factors with regard to DC were analyzed using Chi-Square and logistic regression analysis. Functional outcome was assessed by the extended Glasgow outcome scale one year after the bleeding.

**Results:**

During the study period, 1037 patients were treated for aSAH. Thirty-five patients (3.4%) underwent DC. At one year follow-up, 25 of these (71%) had unfavorable functional outcome. Multivariate logistic regression analysis revealed that poor clinical grade before aneurysm treatment, middle cerebral artery (MCA) aneurysm, edema on the initial computed tomography (CT), and adverse events during aneurysm occlusion were independent and significant risk factors for performing DC.

**Discussion and conclusion:**

DC is relatively uncommon in aSAH patients and is related to increased risk of unfavorable outcome. However, favorable functional outcome was seen in 29% of patients with DC. Adverse events during aneurysm occlusion were significant risk factors for DC.

## Highlights

• 3.4% of patients treated for aneurysmal SAH were operated with decompressive craniectomy.
• Risk factors for DC were poor clinical status, MCA aneurysm, edema, and an adverse event during aneurysm treatment.
• 29% of patients operated with DC had favorable outcome at one year follow-up.
• Age was a significant factor in regards to outcome for patients operated with DC.

## 1 Introduction

Aneurysmal subarachnoid hemorrhage (aSAH) is a severe condition with a high risk of an unfavorable functional outcome (Huhtakangas et al., 2015; Roquer et al., 2020). Despite improvements in surgical and endovascular techniques as well as in neurointensive care, both disease- and treatment-related adverse events are common and affect outcome (Baldvinsdóttir et al., 2023a, 2023b; Muirhead et al., 2021). Decompressive craniectomy (DC) may be necessary to treat intractably high intracerebral pressure (ICP), often caused by intracerebral hematoma (ICH), cerebral infarction, and/or cerebral edema (Pinto et al., 2023; Dunn, 2002). Conservative treatment regimens of elevated ICP in aSAH patients include elevating the head of the bed, reducing fever, treating infections, hyperglycemia and epileptic seizures, optimizing blood sodium levels, draining cerebrospinal fluid, osmotherapy, hyperventilation and increasing sedation (Dunn, 2002; Schizodimos et al., 2020). Decompressive craniectomy is the last option when other methods are insufficient and is often a life-saving procedure (Schizodimos et al., 2020; Jaeger et al., 2003; Sahuquillo and Dennis, 2019; Björk et al., 2023). However, patients with aSAH who undergo DC generally have a poor prognosis (Björk et al., 2023; Alotaibi et al., 2017; Jabbarli et al., 2017).

In the literature, there are widely varying reports on the use of DC following aSAH. A systematic review from 2020 reported a mean frequency of 10.9% with a range from 3.3% to 25.6% (Darkwah et al., 2020). In this study we aimed to examine the use of DC in a cohort of aSAH patients, using a comprehensive database of patients with non-traumatic SAH treated at the neurosurgical centers across Sweden. No studies have been published on this group of patients using a nationwide, unselected sample.

## 2 Methods

### 2.1 Study population and data collection

We used information from a prospective nationwide database from the six neurosurgical centers in Sweden treating patients with aSAH from September 2014 to March 2018 (3.5 years). Located at the university hospitals in Gothenburg, Linköping, Lund, Stockholm, Umeå and Uppsala, these centers covered the entire Swedish population with regard to treatment for aSAH. Data registration was performed in Microsoft Access® (Microsoft, Redmond, WA). A steering group with representatives from all centers assembled regularly to assure that the study protocol was accurately followed. It was decided during the design of the study that any comparison between centers would not be performed.

### 2.2 Clinical and radiological variables

All clinical variables were defined by the steering group prior to initiation of the study. The following clinical baseline parameters were analyzed: age, sex, body-mass index (BMI), pre-existing comorbidities such as previous stroke, coronary heart disease, hypertension, diabetes, use of alcohol, and cigarette smoking. Neurological status prior to aneurysm occlusion (graded according to the World Federation of Neurosurgical Societies; WFNS)(Report of World Federation of, 1988), pupillary response, and focal neurological deficits were noted. Initial radiological findings, such as severity of the bleeding according to the Fisher scale (Fisher et al., 1980), hydrocephalus, cerebral edema, cerebral infarction and angiographic features of the aneurysm such as location, type (saccular, blister, dissecting, fusiform or mycotic), size and neck configuration were also registered. A wide neck was defined as neck size ≥ 4 mm and/or dome-to-neck ratio < 2 (Hendricks et al., 2019). The type of aneurysm occlusion (microsurgical or endovascular) was registered, as well as peri-operative adverse events related to the aneurysm occlusion. These included intraoperative aneurysm re-rupture (Baldvinsdóttir et al., 2023a), temporary parent artery occlusion for more than 5 min (Baldvinsdóttir et al., 2023a; Kameda et al., 2020), and/or adjacent vessel injury for patients treated with microsurgical aneurysm occlusion (Baldvinsdóttir et al., 2023a). For patients treated with endovascular approach; perioperative thromboembolism, perioperative re-rupture, and/or vessel dissection were registered as treatment-related adverse events (Baldvinsdóttir et al., 2023b). We also recorded whether the patients were sedated with thiopental during the intensive care (which may be administered to treat severe edema and/or elevated ICP) and if they developed delayed ischemic neurological deficits (DIND), as previously defined (Al-Tamimi et al., 2010).

Decompressive craniectomy was performed at the discretion of the attending neurosurgeon, most often a standard hemicraniectomy. Criteria for when and how DC was performed were not defined in the study protocol. DC was considered early when performed within 24 h from the time of bleeding and late when performed after 24 h.

### 2.3 Outcome

Functional outcome was assessed one year after the bleeding, with the extended Glasgow outcome scale (GOSE)(Jennett and Bond, 1975) using a standardized questionnaire (Wilson et al., 1998) during an outpatient visit or telephone interview. The results were dichotomized into unfavorable outcome (GOSE 1–4, i.e., dead, or dependent on assistance of others) or favorable outcome (GOSE 5–8, i.e., able to live independently).

### 2.4 Statistical analyses

Chi-square test was performed to identify differences in the profile of clinical variables between patients operated with DC and patients not operated with DC. Univariate and multivariate logistic regression analyses were used to analyze possible risk factors for DC and for unfavorable functional outcome. The number of factors included in each multivariate analysis were limited to one for every ten patients. Factors were chosen based on clinical relevance and the results in the univariate analysis. A probability value of <0.05 was considered statistically significant. Odds ratio (OR) and 95% confidence interval (CI) were calculated through logistical regression. IBM SPSS® Statistics version 28 (IBM Corp., Armonk, NY) was used for the statistical analyses.

### 2.5 Ethics

The Regional Ethical Review Board in Stockholm granted permission to conduct the study (2014/990–3). Informed consent to participate was obtained either from the patients or their next of kin.

## 3 Results

### 3.1 Patient characteristics

In total, 1037 patients were treated for a ruptured aneurysm during the study period. The patients median age was 58 years (range: 6–91). Five patients were younger than 18 years. Three-hundred and twenty-two patients (31%) underwent microsurgery, and 715 patients (69%) received endovascular treatment to occlude the ruptured aneurysm. Of all patients, 35 (3.4%) were operated with DC. Of the 322 patients treated with microsurgery, 24 patients (7.5%) were operated with DC. Of the 715 patients treated by endovascular approach, 11 patients (1.5%) were operated with DC. The DC was performed at a median of 2 days after the bleeding (range: 0–14). Chi-square tests revealed that DC was significantly more often performed if the patients were in a poor initial clinical grade (p < 0.01), had a Fisher grade 4 bleeding (p < 0.01), had aneurysm on the middle cerebral artery (MCA; p < 0.01), had been treated with microsurgery (p < 0.01), had infarction (p < 0.01), and/or edema (p < 0.01) on the initial CT, or if an ICH was evacuated (p < 0.01). Multivariate logistic regression analyses (Table 1) showed that poor WFNS grade (OR 7.25, 95% CI 2.41–21.8, p < 0.01), aneurysm location on MCA (OR 2.86, 95% CI 1.26–6.49, p < 0.01), edema on initial CT (OR 3.62, 95% CI 1.59–8.26, p < 0.01), and adverse events during aneurysm treatment (OR 3.61, 95% CI 1.58–8.26, p < 0.01) were significant independent risk factors for DC being performed.

**Table 1:** Logistic regression analysis. Patients operated with decompressive craniectomy (n = 35).

	Univariate		Multivariate	
	OR (95% CI)	*p* value	OR (95% CI)	*p* value
Age (older)	0.99 (0.96–1.01)	0.25		
Female sex	0.92 (0.45–1.87)	0.82		
History of smoking	1.40 (0.66–2.98)	0.38		
Alcohol	1.36 (0.58–3.19)	0.49		
Diabetes	1.37 (0.32–5.90)	0.67		
Coronary heart disease	2.03 (0.76–5.39)	0.16		
Hypertension	1.40 (0.71–2.78)	0.33		
Previous stroke	0.69 (0.09–5.20)	0.72		
BMI (higher)	0.99 (0.93–1.07)	0.92		
Poor clinical grade (WFNS 4–5)	8.84 (3.39–23.1)	<0.01	7.25 (2.41–21.8)	<0.01
Aneurysm location: ACA/ACoA	0.47 (0.21–1.05)	0.07		
Aneurysm location: ICA	0.61 (0.25–1.49)	0.28		
Aneurysm location: MCA	5.11 (2.58–10.1)	<0.01	2.86 (1.26–6.49)	0.01
Aneurysm location: VA/BA	0.29 (0.07–1.22)	0.09		
Aneurysm size (larger)	1.01 (0.97–1.04)	0.71		
Aneurysm occluded with microsurgery	5.15 (2.49–10.7)	<0.01		
Wide neck aneurysm	1.68 (0.77–3.67)	0.19		
Fisher grade 4	5.12 (2.11–12.4)	<0.01		
Infarction on initial CT	4.37 (1.72–11.1)	<0.01		
Edema on initial CT	7.35 (3.46–15.6)	<0.01	3.62 (1.59–8.26)	<0.01
Hydrocefalus on initial CT	0.97 (0.49–1.92)	0.92		
ICH evacuated	9.34 (4.41–19.8)	<0.01		
DIND	0.99 (0.44–2.22)	0.98		
Adverse events during aneurysm occlusion	3.95 (1.99–7.84)	<0.01	3.61 (1.58–8.26)	<0.01

BMI, body mass index; WFNS, World Federation of Neurosurgical Societies; ACA, anterior cerebral artery; ACoA, anterior communicating artery; ICA, internal carotid artery; MCA, middle cerebral artery; VA, vertebral artery; BA, basilar artery; CT, computed tomography; ICH, intracerebral hematoma; DIND, delayed ischemic neurological deterioration.

### 3.2 Early or late decompressive craniectomy

Of the 35 patients operated with DC, 15 patients were operated within the first 24 h from the time of bleeding (early DC). Of those, eight patients were operated with DC in the same session as the aneurysm was surgically occluded (8/322; 2.5%). Seven of the 15 early DC patients had an adverse event when the aneurysm was occluded. The remaining 20 patients were operated after more than 24 h from the time of bleeding (late DC). Nine of the 20 late DC patients had a complication related to the aneurysm occlusion. Table 2 shows comparisons between the two groups of patients operated with DC, early vs. late. Patients subjected to early DC had more often Fisher grade 4 bleeding (p = 0.02), an intracerebral hematoma evacuated (p = 0.04) and had a higher mortality (p = 0.04). All deaths occurred within 30 days, i.e., there were no additional mortalities between one month and one year among the DC patients.

**Table 2:** Comparison of patients operated with decompressive craniectomy within 24 h from bleeding (early DC) and patients operated later (late DC).

	Early DC (n = 15)	Late DC (n = 20)	Chi-square test *p* value
Poor clinical grade (WFNS 4–5)	13 (87%)	15 (75%)	0.44
Fisher grade 4	15 (100%)	14 (70%)	0.02
ICH evacuated^a^	8 (53%)	4 (20%)	0.04
MCA aneurysm	9 (60%)	10 (50%)	0.56
EVD^b^	14 (93%)	19 (95%)	0.83
Adverse events during aneurysm occlusion	7 (47%)	9 (45%)	0.60
Thiopental sedation	5 (33%)	10 (50%)	0.32
Favorable outcome	3 (20%)	5 (25%)	0.93
Deceased within 30 days	7 (47%)	3 (15%)	0.04
Deceased within 1 year	7 (47%)	3 (15%)	0.04

WFNS, World Federation of Neurosurgical Societies; ICH, intracerebral hematoma; MCA, middle cerebral artery; EVD, external ventricular drainage.

a ICH was evacuated during the microsurgical intervention.

b EVD was inserted before DC.

### 3.3 Functional outcome

Of all patients undergoing aneurysm occlusion, 334 patients (32%) had unfavorable functional outcome (GOSE 1–4) at one year follow-up. Decompressive craniectomy was not an independent risk factor for unfavorable outcome. Multivariate logistic regression analysis (Table 3) showed that increased age (OR 1.05, 95% CI 1.03–1.06, p < 0.01), previous stroke (OR 3.22, 95% CI 1.38–7.55, p < 0.01), poor initial clinical grade (WFNS 4–5; OR 4.41, 95% CI 3.05–6.36, p < 0.01), infarction on initial CT (OR 3.96, 95% CI 1.85–8.47, p < 0.01), edema on initial CT (OR 1.90, 95% CI 1.12–3.23, p = 0.02), hydrocephalus on initial CT (OR 2.32, 95% CI 1.60–3.37, p < 0.01), if ICH was evacuated (OR 3.87, 95% CI 1.53–9.75, p < 0.01), and if there was an adverse event during aneurysm occlusion (OR 1.84, 95% CI 1.17–2.90, p < 0.01) were all independent and significant risk factors for unfavorable outcome at one year follow-up.

**Table 3:** Logistic regression analysis. Patients treated with aneurysm occlusion having unfavorable outcome (n = 334).

	Univariate		Multivariate	
	OR (95% CI)	*p* value	OR (95% CI)	*p* value
Age (older)	1.05 (1.04–1.06)	<0.01	1.05 (1.03–1.06)	<0.01
Female sex	0.88 (0.66–1.81)	0.40		
History of smoking	1.38 (1.04–1.82)	0.03	0.37 (0.07–1.94)	0.24
Alcohol	0.95 (0.66–1.36)	0.77		
Diabetes	3.28 (1.65–6.53)	<0.01	1.46 (0.61–3.51)	0.40
Coronary heart disease	2.94 (1.83–4.71)	<0.01	0.95 (0.49–1.83)	0.88
Hypertension	1.54 (1.17–2.03)	<0.01	0.96 (0.66–1.39)	0.83
Previous stroke	4.72 (2.39–9.32)	<0.01	3.22 (1.38–7.55)	<0.01
BMI (higher)	1.01 (0.98–1.04)	0.43		
Poor clinical grade (WFNS 4–5)	6.81 (5.02–9.23)	<0.01	4.41 (3.05–6.36)	<0.01
Aneurysm location: ACA/ACoA	0.82 (0.62–1.09)	0.17		
Aneurysm location: ICA	0.77 (0.56–1.06)	0.11		
Aneurysm location: MCA	1.41 (1.01–1.96)	0.04	1.04 (0.63–1.70)	0.88
Aneurysm location: VA/BA	1.31 (0.92–1.87)	0.14		
Aneurysm size (larger)	0.99 (0.97–1.01)	0.20		
Wide neck aneurysm	0.91 (0.67–1.22)	0.52		
Fisher grade 4	3.10 (2.33–4.11)	<0.01	1.29 (0.89–1.87)	0.18
Infarction on initial CT	4.51 (2.48–8.21)	<0.01	3.96 (1.85–8.47)	<0.01
Edema on initial CT	4.66 (3.08–7.04)	<0.01	1.90 (1.12–3.23)	0.02
Hydrocephalus on initial CT	3.17 (2.39–4.21)	<0.01	2.32 (1.60–3.37)	<0.01
ICH evacuated	6.55 (3.48–12.3)	<0.01	3.87 (1.53–9.75)	<0.01
DIND	2.55 (1.86–3.49)	<0.01	1.13 (0.32–3.97)	0.85
Adverse events during aneurysm occlusion	1.57 (1.11–2.20)	0.01	1.84 (1.17–2.90)	<0.01
Decompressive craniectomy	5.58 (2.49–12.5)	<0.01	2.88 (0.90–9.23)	0.08

BMI, body mass index; WFNS, World Federation of Neurosurgical Societies; ACA, anterior cerebral artery; ACoA, anterior communicating artery; ICA, internal carotid artery; MCA, middle cerebral artery; VA, vertebral artery; BA, basilar artery; CT, computed tomography; ICH, intracerebral hematoma; DIND, delayed ischemic neurological deterioration.

Of the 35 patients operated with decompressive craniectomy, 25 patients (71%) had unfavorable functional outcome. Patients that were operated with DC and had unfavorable outcome were significantly older (median age 57 years; p = 0.02; binary logistic regression) than those who had favorable outcome (median age 49 years). Other factors such as WFNS grade, aneurysm location on MCA, edema on initial CT, and adverse events during aneurysm occlusion were not significant in this regard. As seen in Table 2, all DC patients that died did so within 30-days following the bleeding.

## 4 Discussion

In this prospective nationwide study of an unselected cohort of patients treated for aSAH, the ratio of patients subjected to DC was 3.4%. In the literature, variable frequencies of DC following aneurysmal SAH have been described, ranging from 3.3% to 25.6% with a mean ratio of 10.9% (Darkwah et al., 2020). There are no international guidelines on when to perform DC in aSAH patients.

The rate of DC was 7.5% for microsurgical cases and 1.5% for endovascular cases. Such a difference has previously been described in the literature (Darkwah et al., 2020; Güresir et al., 2009; Jabbarli et al., 2020) and may, at least partly, be explained by relatively more MCA aneurysms and ICH among the patients that underwent microsurgery, factors related to increased risk of DC. Furthermore, in our cohort 18% of patients treated with microsurgery had cerebral edema on the initial CT compared to 11% of those treated with endovascular approach (Baldvinsdóttir et al., 2023a, 2023b).

### 4.1 Risk factors for DC in patients with aneurysmal SAH

Patients with aneurysms on the MCA and patients in poor clinical grade were significantly more likely to be operated with DC, as seen in the multivariate analysis. This agrees with what has been described (Güresir et al., 2009; Buschmann et al., 2007; Brandecker et al., 2021). In addition, our study shows that patients with edema on the initial CT and those having an adverse event during aneurysm occlusion procedure were more likely to have DC. We have recently analyzed adverse events during microsurgery and endovascular treatment of ruptured aneurysms and their impact on outcome (Baldvinsdóttir et al., 2023a, 2023b). Their effect on the increased risk of having to perform a DC further emphasize the severity of adverse events during aneurysm occlusion. A systematic review from 2020 (Darkwah et al., 2020) showed that a younger patient age was a risk factor for DC being performed. We did not see this as a factor in our study.

As the patients operated with DC were relatively few (n = 35), limited number of variables could be selected for the multivariate analysis. Several factors were significantly associated with DC in the univariate analysis but only four were entered in the multivariate analysis. We selected factors that we considered the most clinically relevant for predicting DC. Poor clinical grade (WFNS 4–5) prior to aneurysm occlusion, aneurysm location on MCA, edema on the initial CT, and adverse events during aneurysm occlusion were independent significant risk factors. Furthermore, the results for Fisher grade 4, infarction on initial CT, and evacuation of ICH in the univariate analysis emphasize the importance of the severity of the aSAH as a risk factor for the need for DC.

At the Swedish centers, DC is performed when other measures to treat elevated ICP fail. These are primarily drainage of cerebrospinal fluid through an external ventricular drain and a stepwise increase in sedation. The DC operations are not performed prophylactically, i.e., before a severe ICP problem arises, with DC there becomes risk of further surgical adverse events/complications and need for future cranioplasty (Kurland et al., 2015). A recent study from Uppsala (Ronne-Engström et al., 2024) showed a decreasing trend in the number of DCs performed at their clinic in recent years, possibly reflecting improved neurointensive care.

There were no major differences in clinical status or initial radiological findings of patients who were operated on within 24 h (early) or after 24 h (late). Forty-three percent of patients undergoing DC were operated within the first 24 h after the bleeding. These patients were more likely to be deceased at one-year follow-up than patients operated later. We theorize that the primary impact of the bleeding and the resulting edema is more severe and may have different underlying pathophysiology than the edema occurring later in the clinical course. However, this should in our opinion not exclude patients from undergoing surgery within 24 h. The lower proportion of patients who underwent DC in our study (3.4%) compared to the mean ratio described in the literature (10.9%) could be explained by differences in medical practice. Centers may differ in their criteria for active treatment of patients in poor clinical grade. Thus, patients in a very poor clinical condition later during the neuro-intensive care might have been subjected to withdrawal of care instead of being operated with DC.

### 4.2 Functional outcome after DC

Of all patients treated for aSAH during the study period, 32% had unfavorable functional outcome (GOSE 1–4) one year after the bleeding. Of patients operated with DC, 71% had unfavorable outcome. On the other hand, 29% of patients operated with DC following aneurysmal SAH had favorable functional outcome. This might be encouraging considering the often poor clinical condition of these patients. Similar results were reported by Güresir et al. (Güresir et al., 2009) where 26% of patients operated with DC had favorable functional outcome (modified Rankin Score (mRS) 0–3) after six months. In their study the frequency of DC following aSAH was 5.5%. A study by Jabbarli et al. (2020) showed that early DC was associated with higher rates of favorable functional outcome compared to DC being performed later. In that study early DCs were performed in the same session as the aneurysm was microsurgically occluded or directly following endovascular treatment. This suggests a more aggressive operative approach for treating cerebral edema than in our study.

The strength of this study is the prospective and complete nationwide registration of patient information. However, we do not have specific information about the surgical method, such as the size of the craniectomies, incision techniques, postoperative infections. Nor do we have data on ICP recordings. These factors were not within the scope of the study, but of clinical interest and should be investigated further. An interesting development in this regard is the so-called hinge-craniectomy technique, where the bone flap is left in situ but pivoted (Omerhodzic et al., 2023).

## 5 Conclusion

Decompressive craniectomy was performed in a relatively low number of patients with aSAH (3.4%), the majority of whom experienced an unfavorable outcome. The main risk factors for the need for DC were poor clinical grade, aneurysm location on the MCA, edema on initial CT, and adverse events during the occlusion of the ruptured aneurysm. Younger patients subjected to DC had a higher chance of favorable outcome and 29% of all patients who underwent DC returned to independent living. Our findings support that DC is a valuable tool in treating the poor-grade or deteriorating patient with aSAH. While younger patients seem to benefit more from DC, older patients may also have a favorable outcome after the procedure.

## Contributorship statement

Bryndís Baldvinsdóttir MD: Data collection, Data analysis and interpretation, Drafting the article, Critical revision of the article, Approval of the submitted article, Corresponding author, Writer of the cover letter.

Erik Kronvall MD PhD: Data collection, Data analysis and interpretation, Drafting the article, Critical revision of the article, Approval of the submitted article.

Elisabeth Ronne Engström MD PhD: Designing the study database, Data collection, Critical revision of the article, Approval of the submitted article.

Per Enblad MD PhD: Designing the study database, Data collection, Critical revision of the article, Approval of the submitted article.

Peter Lindvall MD PhD: Designing the study database, Data collection, Critical revision of the article, Approval of the submitted article.

Helena Aineskog MD: Data collection, Critical revision of the article, Approval of the submitted article.

Steen Friðriksson MD: PhDDesigning the study database, Critical revision of the article, Approval of the submitted article.

Paula Klurfan MD: Data collection, Critical revision of the article, Approval of the submitted article.

Mikael Svensson MD PhD: Designing the study database, Data collection, Critical revision of the article, Approval of the submitted article.

Peter Alpkvist MD: Data collection, Critical revision of the article, Approval of the submitted article.

Jan Hillman MD PhD: Designing the study database, Data collection, Critical revision of the article, Approval of the submitted article.

Johanna Eneling MD: Data collection, Critical revision of the article, Approval of the submitted article.

Ola G. Nilsson MD PhD: Designing the study database, Data collection, Data analysis and interpretation, Drafting the article, Critical revision of the article, Approval of the submitted article.

## Declaration of competing interest

The authors declare that they have no known competing financial interests or personal relationships that could have appeared to influence the work reported in this paper.
